# Zoonomia: Or the Law of Animal Life

**Published:** 1853-05

**Authors:** Marshall Hall

**Affiliations:** Foreign Associate of the “Académie de Médecine” of Paris, &c. &c.


### MEDICAL EXAMINER,

AND

### RECORD OF MEDICAL SCIENCE.

NEW SERIES.—NO. CI.—MAY, 1853.

### ORIGINAL COMMUNICATIONS.

*Zoonomia: or the Law of Animal Life. A Lecture delivered at the Smithsonian Institute, Washington, U. S., on March the 8th*, 1853. By Marshall Hall, M. D., F. R. S., Foreign Associate of the "Académie de Médecine" of Paris, &c. &c.

Gentlemen,—I congratulate myself on the opportunity afforded me, by your kind invitation, to unfold a principle in physiology on which I have long meditated and experimented, and which I have ventured to designate the Law of Animal Life.

Many have been the attempts to define *life*. All have, I think, proved abortive. I shall not, in my turn, venture to attempt that in which so many have failed. My object will be rather to describe than to define.

Life, then, may be justly viewed as consisting in a comprehensive system of action and re-action—of the action of certain physical and chemical influences, and the re-action of certain vital powers in organized beings.

Throughout the animal, and, indeed, the vegetable kingdoms, the *primary* organic agents and re-agents are *pollen* and *ova.* Life first consists in the reciprocal action of these upon each other. Such is the very type and essence of life in its earliest dawn. Harvey said—Omne vivum ab *ovo.* He might, with equal truth, have said, Omne vivum a *polline;* and with still greater truth, Omne vivum a polline *et* ovo.

Each of them—the pollen, the ovum—was originally a *creation.* Their mutual and reciprocal action is a phenomenon which the Creator has impressed on this portion of His works, as the *fiat* of His will.

That this action *is* perfectly reciprocal, is proved by the resemblance of the new being or offspring—be it animal or vegetable—to *both* parents. The event is at present as inscrutable in its nature and essence, as it is interesting to the physiologist and philosopher as a subject of observation and new inquiry.

Why *this* seed of the Triticum Indicum, or Indian corn, on which pollen has fallen, should, if planted in soil and exposed to the genial influences of heat and moisture, become a noble and useful plant; and this other, to which, from its mode of growth and treatment, no pollen has been allowed to have access, shall, under similar circumstances and influences, undergo decay and decomposition, who can tell? Why *this* egg, which has been fertilized by pollen, should, under similar genial influences, become a bird, and eventually soar into the regions of the atmosphere; and why this other, unaffected by pollen, should pass into a state of putrefaction, who can declare? Who will attempt to explain why those "divinæ particulæ auræ," which exist in the form of pollen, can alone vivify these ova? Who can say *what* there is in these ova, and *what* there is in the appropriate pollen—for the *two* are *equally* essential—which develope growth, and form, and *life?*

Look at these ova and on these seeds. On *this* ovum and on *this* seed no pollen has ever been shed. They will, though placed in circumstances the most favorable for developement, only pass the more readily into decay. But this other ovum, this other seed, on which pollen, life-giving pollen, has shed itself, will, under similar circumstances, germinate and pass into life, the whole subsequent being being expressly imbued with the equally inscrutable properties of the pollen, and of the ovum or seed.

What is the condition of this pollenized, this fructified ovum or seed *before* active life begins? Is life in abeyance? or is it in actual existence only in its lowest and imperceptible form? Have eggs and seed a temperature of their own, above that of the medium in which they are placed? And what is the condition of this *offset* of an animal or animalcule, (as the planaria,) or of this plant? Both will continue to live independently of the original stem. Both may be multiplied and propagated by new and similar offsets. Both are, as I have stated, equally imbued with the properties, both of the original pollen and ovum. All this is mysterious, inscrutable in its essence, constituting one of the arcana of nature, which may long, may forever be hidden from us.

But the *laws* of life, and those of the material and inorganic world, may be detected, and their detection and investigation are amongst the most legitimate, interesting and important objects of philosophical inquiry. At present I beg only to state, that whilst pollen places the ovum or seed under *vital* influences, its absence allows them to become the prey of mere physical or chemical agencies alone.

The further essential and distinctive characteristic of organized beings, that in which they differ from the objects of the inorganic world, is—*membrane*. Through this membrane, a special function of transition takes place, constituting endosmosis and exosmosis, or imbibition and exudation.

If this membrane be injured, or broken down, the materials of organized beings are again immediately subtracted from the laws of organization, and delivered over to the ordinary principles of chemical action—decay, or eremacausis—from which that membrane had preserved them, whilst it placed them under the dominion or modification of vital influences. Examples of this fact are afforded by bruises of animal or vegetable tissues, which, if slight, are repaired by the vital powers; but if severe, lead to death or decay. How extraordinary are the changes which almost immediately take place in bruised flesh, or in a bruised orange or apple!

But I must hasten from these preliminary views, and pass on to the more express subject of this lecture. All living beings, from the serpent to the eagle, possess, in common, peculiar *dynamic* properties ; in all, these properties respond to appropriate external and internal *stimuli.* On the reciprocal play, action and re-action, of those forces and agents, life, with all its varied phenomena, in all its varied forms, essentially depends.

These dynamics and these stimuli bear a relative proportion to each other; this proportion is *inverse*; the higher the dynamic, the lower the stimuli, and *vice versa. Such is—the Law of Animal Life.*

In the animal kingdom, *two* forms of dynamics exist; the first has its seat in the nervous system, or, more definitely, in the spinal and ganglionic sub-systems; I venture to designate it by the term *neuro-dynamic.* The second has its seat in the muscular system ; it may be designated *myo-dynamic.* The stimuli exist in greater numbers, for they consist in physical and chemical agents of the external world—such as air, food, water, heat, light, the galvanic influence, &c.

I must introduce the subject of the vital dynamics by showing those who are not familiar with physiology, an experiment full of the deepest interest; I place before you a frog, prepared for the purpose, and you will observe how the most elevated principles of philosophy may be illustrated by what you may deem the humblest objects of creation. The physiologist knows and feels that his science, elevated as it is, is included in the most insignificant insect that lives—the caterpillar or the butterfly.

The nervous system consists in *three* sub-systems; the first is that in which the brain, or cerebrum, is the centre, and sensation and volition are the special functions ; I have removed this centre and these functions from this frog, by removing the head, and, with it, the very centre of the system, or sub-system, itself; the creature is thus entirely deprived of sensibility; the idea of suffering is excluded. It is also deprived of volition—all *spontaneous* movements are impossible.

Yet there *is* a source and power of movement remaining, for you observe the effect of irritation of the integument covering the toe or foot. This power resides in the spinal marrow, or spinal centre, and certain nerves proceeding *to* it and *from* it, termed therefore, eisodic and exodic. I have designated this power neurodynamic; it was formerly termed the *vis nervosa*; its English appellation would be *nerve*-power.

The nerves which proceed *to* the centre of this spinal system, *arise* from the skin. I have removed them from the left foot, and you will observe that the same irritation which, applied to the right foot, induced contraction of the muscles, is inoperative when applied to this wounded limb; in effect, the *origin* of the eisodic nerve has been removed with the integument.

But I now irritate the spinal centre itself: you observe the convulsions produced.

Lastly, I irritate these, the lumbar nerves; they are exodic, and proceed to the muscles: again the limbs are violently agitated by movements.

These, then, are phenomena arising out of irritation of the nerves, eisodic and exodic, and the centre of the spinal system. They attest the neuro-dynamic power of those several nervous tissues.

But there is still a third sub-system of the nervous system; it is termed the ganglionic, and it is connected with all that is *interior*, or *within* us. I have here placed aside the viscera of the frog, and with them the ganglionic sub-system belonging to them—the heart, the stomach, the intestines, &c. If you were nearer, you would see the heart pulsate, and the stomach and intestines move by what is termed peristaltic action. These phenomena are effectible through the medium of the ganglionic portion of the nervous system, in which the neuro-dynamic must also reside.

But besides the power residing in the nervous tissues, there is, as above stated, another dynamic. Its seat is the *muscular* tissue; for which reason it may be designated the *myo-dynamic*. You observe the effect of a very slight galvanic influence passed along this muscle; the muscle is immediately vigorously contracted. This is the vis muscularis of physiologists, the *muscle*-power.

I must again hasten away from this explanation. I repeat that these vital dynamics, and the physical and chemical stimuli to which I have alluded, bear an inverse ratio to each other. This is the case both primarily and secondarily; the first, by creation; the second, by a natural operation and *effect;* for if stimulus be diminished, the dynamic becomes augmented: and if the stimulus be augmented, the dynamic becomes exhausted, and, in some degree, proportionately reduced, as natural events, causes and effects. Thus the effect of hibernation, during which the stimuli of air, food, temperature, and nutrition, are reduced to their minimum, is to lead to augmented dynamic and excitability, and to what may very appropriately be designated, *vernation*, or the activity of spring; whilst the effect of the augmented stimulus in the summer months, that is of augmented air, food, temperature, and nutrition, is to exhaust or lessen the dynamic of nervous and muscular fibre, however they may augment general activity and power of mass, and prepare the way for the next winter's sleep.

By creation, and the operation of natural causes, then, the inverse ratio between dynamic and stimulus, in animated creation, is—the Law of Life.

The attempt to invert this law in either direction, and *equally* in either direction, is to destroy life. Unduly to augment the stimulus when the dynamic is high; or unduly to diminish the stimulus when the dynamic is low, is equally to interrupt the vital actions.

I will again illustrate my subject by a reference to the interesting case of hibernation:—If you take a bat from its winter quarters, from its state of hibernation, in which its respiration is at the minimum, and its dynamic at the maximum, and make it fly about, and so augment the vital stimulus of respiration, it infallibly *dies!* If, on the other hand, you take the same creature in its condition of summer activity and of high respiration and low dynamic, and deprive it of air, by immersion in water or in an irrespirable gas, it dies, too. Invert in either way the inverse ratio of dynamic and stimulus, and the result is fatal.

Low dynamic *requires* high stimulus; high dynamic, low stimulus. The higher the dynamic, the more capable is the animal of the further abstraction of stimulus, and *vice versa.* If, instead of taking a bat from its summer activity, you take it in its state of hibernation, and now immerse it in water for ten minutes, or even longer, it is altogether uninjured. The bat taken in its state of activity, and submerged in water, dies in two minutes and a half.

Thus the hibernating animal dies if its respiration be augmented whilst it can bear its suspension ; the same animal, in its state of vernation, or of activity, can bear its respiration to be augmented, but dies speedily if it be suspended !

I will illustrate this view by another order of facts :—the tadpole of the frog breathes in water, and feeds on water plants ; the same tadpole becomes a frog, breathes in atmospheric air, and feeds on insects ; it has become a higher breather—a higher feeder. In the former state, the dynamic, in the latter, the stimulus, is comparatively greater. The tadpole would die if taken out of its element, the water ; the young frog would drown if compelled to remain in it !

These facts are the results of innumerable experiments. I shall take occasion to revert to them hereafter.

Besides being *inverse*, to which there is no exception, the *ratio* between dynamic and stimulus may be higher or lower. It is in this manner that we are enabled to explain the *modes* of life. As life in general is a result of stimulus *into* dynamic, we should, without a provision of this kind, see all animals *equally* active or inactive. Either the reptile would not creep slowly, or the bird tribe would not soar into the atmosphere. But we observe, in fact, that when the stimulus is *dis*-proportionately low, the animal is of low activity ; and that when it is *dis*-proportionately high, the animal is in the enjoyment of an intense degree of activity.

Throughout animated nature, as I have already stated, in all the varied forms and modes of life, from the eagle to the serpent, the dynamic and the stimuli are in an inverse ratio to each other. Such, as I have observed and repeated, is the law of life. In the bird-tribes, the quantity of air and food imbibed is extreme, the degree of dynamic very low ; in the reptile tribes, the quantity of stimulus is low, and the degree of dynamic high. The following formulæ may serve to express this general fact :

| | | | | |
|---|---|---|---|---|
| *Stimulus,* | 8 | 4 | 2 | 1 |
| *Dynamic,* | 1 | 2 | 4 | 8 |

The degree of activity, or of inactivity, in all these cases may be supposed to remain the same.

But to explain the greater activity of the bird, and the inactivity of the reptile, a modification of the formulæ is required, which may be thus expressed :

| | | | | |
|---|---|---|---|---|
| *Stimulus* | 1 | 2+1 | 4+2 | 8+4 |
| *Dynamic* | 8 | 4 | 2 | 1 |

In this manner, whilst the inverse ratio between the stimulus and the dynamic, generally speaking, remains, that of the former may *augment* more rapidly as we pass into the more active forms of living beings than that of the latter *diminishes*; and thus the bird and the insect fly, whilst the reptile and caterpillar creep. With higher stimulus, the animal becomes more bird-like; with lower stimulus, it becomes more reptile.

With augmented air and food, other organs besides those of respiration and digestion become stimulated to greater action. There is especially a correlation between the rapidity of the action of the heart and of the acts of respiration arising in a peculiar and reciprocal manner out of the play of stimulus and of the neuro-dynamic which resides in the spinal and ganglionic sub-systems, and the myo-dynamic in the muscles which are respectively under their dominion, and out of the law which binds them together, which deserves to be distinctly described: The blood flowing through the lungs exhales carbonic acid; this is the internal excitor of inspiration acting on the fine branches of the pneumogastric nerves spread over the lining membrane of the lungs; the more rapid the circulation, the greater the quantity of carbonic acid exhaled, and consequently the more rapid the respiration. But this respiration brings the oxygen of the atmosphere into contact with the pneumonic blood in its turn, through the same pulmonary membrane; this oxygen is absorbed by the blood, passes into the circulation, and stimulates the heart to augmented action, and augments the rapidity of the circulation. This last has again, in its turn, a greater exhalation of carbonic acid in the lungs, again augmenting respiration; &c. In proportion to the augmented stimulus the dynamic is diminished.

The *changes* which take place in regard to the ratio of dynamic and stimulus are of *two* kinds: 1, *structural;* 2, *physiological.* The former, in metamorphosis, is usually, if not always, upwards, *to* a state of higher activity, to a state of higher stimulus with diminished dynamic; the latter takes place in both directions, being to one of higher stimulus in vernation, and to one of higher dynamic in hibernation. Activity on one hand, and repose, and, especially, sleep, on the other, *induce* similar though less marked effects.

I think I have said enough to convince you, gentlemen, that there is, in this Law of Life, a most interesting and important fact, a vast generalization. This generalization embraces *three* great objects: 1, the scale of animated being; 2, metamorphosis and perhaps mere development; 3, physiological changes. I know of no law so general, so expansive.

I may now observe that it is of deep interest to trace the *criteria* of the ratio between dynamic and stimulus.

In the first place, galvanism is a test of neuro- and myo-dynamic, just as nerve and muscle, in the animal in which these dynamics are high, as the frog, become galvanoscopic or a test of galvanism.

In the second place, in the animal in which stimulus is high, the temperature and its measurer,—the thermometer,—become a test of its degree, and, of course, of the inverse condition of the dynamic.

Thirdly, the degree of activity or of inactivity denotes the relative condition of the two elements of the Law of Life.

Fourthly, it has already been noticed that, in proportion to the dynamic, and in the inverse proportion to the stimulus, the animal possesses the power of bearing the subtraction of stimulus, the privation of air and of food, and is, in more senses than one, endowed with *tenacity of life.* The length of time during which an animal can bear the privation of air, or breathe a given limited quantity of air, is proportionate to the dynamic.

In the fifth place, the quantity of respiration affords a measure of the stimulus. This is ascertained in various ways: 1, by the structure and extent of the *lungs;* 2, by the number of the respirations; 3, by the quantity of oxygen imbibed and of carbonic acid exhaled.

In proportion to the *surface* of the lungs on which the methæmatous or blood-changing channels are spread, in proportion therefore to the complexity of the structure of the lungs, is the quantity of respiration. The fish has a mere gill; the batrachian has a vesicular lung, with or without subdivisions or intersections, as we observe in the triton, or in the frog or toad, respectively; the lung of the serpent, the tortoise, the tribes of the mammalia and of the birds, becomes more and more complex and extended; in the insect and in the bird, the respiration is extended over the system, not being limited to one organ; in the insect, indeed, each articulate segment is furnished with an analogue of the medulla oblongata, as a central nervous organ of the respiration. The dynamic exists in an inverse proportion.

In the sixth place, the quantity of food assimilated or respired is a stimulus in itself, and in its proportion to respiration becomes a measure and criterion of the degree of dynamic inversely. In speaking of the quantity of stimulus as represented by the food, we must bear in mind the quality as well as the quantity of that food, and its convertibility into calorifacient and nutrient principles. *Insect* food is perhaps of all kinds of food the most stimulant, whilst vegetable food is the least so. It must also be a question how much of the food is really made available, and how much is excreted unrespired, unassimilated.

Seventhly, we have in the circulation a criterion of the kind and character of life; slow and with few methæmatous vessels, in the animal of low stimulus and high dynamic, it becomes quicker with more crowded vessels as the stimulus is greater. The structure of the lung and the degree of rapidity of the movement of the blood globules, must be carefully noted: as the former becomes more complicated and the latter augmented, the quantity of stimulus is higher, and, I need scarcely say, the degree of dynamic lower.

There is, indeed, no subject so replete with interest as the circulatory apparatus—pneumonic and systemic—in themselves, in the different orders of animated being, and in reference to the law of life. The entire apparatus consists of—1, the minute arteries; 2, the minute veins; and, 3, the intermediate blood-channels, or, as I have proposed to denominate them—from the important fact that all the changes which take place in the blood take place in them—the methæmatous or blood-changing channels. These vessels are specifically distinct, a distinction on which I have insisted on another occasion.

I must now, Gentlemen, in the last place, bring before you certain *results* of that law of life which I have thus very inadequately sketched. In doing this, I shall be compelled to repeat some of the preceding remarks; but I prefer to do this to the alternative of leaving my sketch incomplete.

The first remark I have to make in regard to the results of the Law of Life, relates to the temperature of animals of high dynamic and of low respiration, and consequently of low temperature. Such animals are said to be of *cold blood.* This expression is inaccurate. No animal is positively of cold blood. The species of lowest temperature is still of a temperature higher than that which would subsist absolutely without respiration, and its blood is only *low* in temperature, without being as cold as the surrounding medium.

Even amongst fishes some are high, others low, feeders and breathers, with a corresponding temperature; the trout can only live at the surface of a limpid stream, breathing highly oxygenated water, and feeding on the insects immediately on that surface; the carp, on the contrary, lives and breathes lowly, at the lowest part of stagnant pools. The trout is, comparatively, a fish of high stimulus—food and respiration—and temperature, and of low dynamic; the carp, of high dynamic and low stimulus. The trout dies almost immediately if taken out of its crystal element; the carp will live for days in wet moss, that is, out of its own element, abundantly supplied with moisture, or in a limited portion of water ill supplied with mixed atmospheric air.

As we rise in the scale of animated being, from the fish to the reptile, from this to the mammalia, and from these to birds, the respiration, and, with this, the temperature, also rises, the dynamic proportionately falling; the temperature of the fish and reptile is slightly above that of the medium in which they dwell respectively; that of the mammalia is about 98°, that of the bird tribes about 102°, Fahr.

The temperature accurately coincides with the quality and quantity of food, the quantity of respiration, and is, in effect, the developement of an internal stimulus from stimulant ingesta.

With temperature there is also probably the evolution of the galvanic agency.

The galvanic apparatus, the thermometer, the quantity of food, the quantity of oxygen, the power to bear the abstraction of these stimuli, or, in certain circumstances, their addition; all these are criteria of the place a given animal, in a given condition, ought to occupy in the zoological or physiological scale.

Growth, development, metamorphosis, nutrition, in ovo and extra ovum, are other results of the play of vital powers, dynamic and stimulus. With each of such changes in form, a change in kind of life, or a metabiosis occurs.

Of these, hints have been dispersed in the preceding remarks. If I have succeeded in giving you, Gentlemen, an adequate idea of them, and of the other topics involved in the development of the Law of Life, I shall feel much gratified. Pray accept my best thanks for your kind attention throughout this imperfect lecture.

Each part of my subject would afford scope for distinct discussion, and one object of my visit to the United States is to secure to myself both the leisure and the opportunity for further physiological inquiry in regard to it. In this object I know and feel that I shall be assisted by the liberal and generous people amongst whom it is my lot, for a time, to dwell, and by the noble and free institutions, the objects of which it may, as on the present occasion, be my proud privilege humbly to promote.

Of the Smithsonian Institute I can only most cordially say, may it prosper, and may it long be the means of the diffusion of knowledge, and of consequent good to mankind!

